# At the Heart of the Problem: Health in Johannesburg’s Inner-City

**DOI:** 10.1186/s12889-017-4344-2

**Published:** 2017-07-04

**Authors:** Helen Rees, Sinead Delany-Moretlwe, Fiona Scorgie, Stanley Luchters, Matthew F. Chersich

**Affiliations:** 1 Wits Reproductive Health and HIV Institute (WRHI), Faculty of Health Sciences, University of the Witwatersrand, Johannesburg, South Africa; 2 Burnet Institute, Melbourne, Australia; 3 Department of Epidemiology and Preventive Medicine, Medicine, Nursing and Health Sciences, Monash University, Melbourne, Australia; 4 International Centre for Reproductive Health, Department of Urogynaecology, Faculty of Medicine and Health Sciences, Ghent University, Ghent, Belgium

Urban life in the twenty-first century is marked by numerous stresses and shocks, resulting from rapid urbanisation, frequent migration and crowding, massive unemployment, climate change, physical disasters, and disease outbreaks, among other challenges. This reality – according to the ‘100 Resilient Cities’ initiative of the Rockefeller Foundation – is why the concept of resilience is critical to a sustainable future: cities must learn to “survive and thrive, regardless of the challenge” [1]. In cities in the global South that face a heavy HIV burden, this health crisis is often inseparable from a wider set of interlinked social challenges, ranging from acute economic inequality to chronic political mismanagement and failed states. What ‘resilience’ means in such contexts is hard to imagine, but at the very least, as UNAIDS’ Michel Sidibé puts it, “people must be at the centre of the response” [2].

This Supplement to *BMC Public Health* brings together articles on the health status and needs of people living in the inner-city of Johannesburg, South Africa, with a particular focus on the impact of the HIV epidemic and on identifying ways to improve healthcare service delivery. At the centre of the inner-city lies Hillbrow, with its dense population of economic migrants and refugees, a neighbourhood that figures in the public imagination as a space virtually synonymous with slums, crime, drug dealing and sex work. Daily life for residents is profoundly shaped by a chronically neglected public infrastructure and lack of investment in human capital. Prevalence of HIV and TB is high, diseases linked to poor sanitation and water quality are common, and morbidity resulting from violence and substance abuse is among the highest in the country [3–5]. By any measure, standards of health and wellbeing among the population of Hillbrow and its surrounding inner-city areas are alarming.

But in terms of healthcare services, this part of the inner-city is, quite inspiringly, a place of ‘many firsts’. Dating back to 1889, the Hillbrow Hospital was the first to be established in the city of Johannesburg, and it was here that nursing was first taught in South Africa as an academic discipline (in collaboration with the Wits Medical School), in the late 1930s [6]. On Hillbrow’s margins, the Charlotte Maxeke Johannesburg Academic Hospital (formerly the Johannesburg General) has been the site of several major clinical milestones in the country, including the first integrated antenatal and ART clinic [7], as well as advances in the fields of renal disease treatment [8], and patient-controlled acute pain relief [9]. The broader community of Hillbrow has seen pioneering developments in the provision of urban-based primary healthcare services [10] and of the first targeted clinical services for sex workers in the country [11]. Today, Hillbrow boasts a high density of healthcare facilities, many of these clustered under the umbrella of the Hillbrow Health Precinct. This development is an exceptional example of how health services can be used to accelerate the rehabilitation of marginal inner-city areas, promoting synergies and progress that would otherwise not be possible.

It is with this background in mind that the editors of this Supplement invited both current and past researchers from the Wits Reproductive Health and HIV Institute (Wits RHI) to contribute papers reporting demographic, epidemiological, ethnographic, and health service data collected in the inner-city over the last ten years. Situated within the Hillbrow Health Precinct, the Institute has collected a wealth of data, both for research purposes and for evaluation of routine health services. The Supplement considers the health needs of the population in this setting, and the challenges of managing HIV (and sexual and reproductive health [SRH] more broadly) among diverse groups: migrants, sex workers, pregnant women, adolescents, and men living with HIV. Critical questions are asked: what are the realities of living in this most notorious pocket of Johannesburg’s inner-city? What are the most pressing health needs? How do we design and deliver effective and sustainable health interventions to these often hard-to-reach populations? What public health lessons may be extracted from the data? Collectively, the findings constitute a valuable resource to inform the design and implementation of cutting-edge, comprehensive HIV and SRH services in inner-city settings. The articles also provide baseline and actionable information for the broader UNAIDS ‘Fast-Track Cities’ initiative’ to end AIDS in cities around the globe [12]. Although Hillbrow is in many senses unique, with a social history intimately linked to Apartheid and its demise, this setting presents a microcosm of issues that are common to other low-income, inner-city areas in southern Africa and beyond. We believe this Supplement will have significance beyond Hillbrow, and indeed, South Africa, as it crystallises many of the public health challenges generic to disadvantaged inner-city neighbourhoods across the globe.

The collection of nine research articles is prefaced by a fascinating social history of Hillbrow assembled by Jonathan Stadler and Charles Dugmore that reveals “a picture of enduring continuities across its 120-odd years of existence”. Today, Hillbrow is largely defined by its transient populations and high proportion of foreign nationals who are new to the city, but historically it has been a space of social change. Its apartment blocks were the site of early racial integration before Apartheid’s segregation laws were relaxed, and the neighbourhood became host to progressive sexual identity movements long before they were embraced elsewhere in the country. These kinds of developments, argue Stadler and Dugmore, have given Hillbrow its peculiar flavour, making it “distinctive in the context of South African urban spaces and even within histories of the post-colonial city more broadly”. This radical flavour is reflected in local responses to HIV: some of the first support groups for people living with HIV formed in Hillbrow in the early years of the South African epidemic, and since then it has been the site of cutting-edge initiatives in HIV prevention, care and treatment.

Hillbrow serves as a portal of entry into the city for many who are searching for new opportunities, a new life. The buzz of its streets crowded with traders, entrepreneurs, sex workers, commuters, students and school children, attest to the cosmopolitanism of this melting-pot neighbourhood. But many migrants are alienated from the system and remain excluded from what the broader city has to offer. With few alternatives available, migrant women may turn to sex work for a living. Johannesburg’s inner-city has for long been known as a place with a high concentration of sex workers, and much has been written over the years about the shifting population of workers in the Hillbrow sex industry, whether female, male or transgender [13–15]. The targeted health services first set out by Wits RHI’s predecessor, the Reproductive Health & HIV Research Unit (RHRU), have been providing HIV and other SRH services for female sex workers and their clients for the last 21 years. Who are the sex workers accessing these services today? And how do they compare – in terms of demographics and reproductive health status – to sex worker populations in other South African cities? Using comparative data obtained from female sex workers in Hillbrow and Pretoria, a nearby city, Mariette Slabbert and colleagues describe the heterogeneity of the former group and its disparate needs. When compared to sex workers in Pretoria, those in Hillbrow had lower levels of HIV, but much higher levels of STIs and frequency of alcohol use; they also tended to be younger, better educated, and more likely to have come from outside South Africa. The majority of Hillbrow sex workers in this study, in fact, had only recently migrated to Johannesburg from Zimbabwe and other neighbouring countries. Their contact with healthcare providers was limited, owing to a combination of stigma and xenophobia. When health services were provided via outreach to their places of work, however, these were accessed far more than services offered in clinical settings. Even then, a number of unmet SRH needs remained: only about 15% were using contraception other than condoms, and current pregnancies were largely unwanted. Female sex workers in Hillbrow, then, experience multiple, overlapping layers of vulnerability – partly because the work they do continues to be stigmatised, but also because of the complex patterns of exclusion generated by their migrant status.

Understandably, much of the concern around xenophobia in South Africa’s urban settings focuses on immediate consequences, such as foreigners’ exclusion from basic services, and violent evictions from their homes. But the consequences are also more long term and systemic. A study of pregnant women’s attendance at three urban antenatal care (ANC) settings in Johannesburg by Siphamandla Gumede et al. found that the Hillbrow facility – a primary healthcare clinic – had the lowest levels of ANC attendance and HIV testing and the highest HIV prevalence of all three. Since the Hillbrow facility serves a clientele of mostly foreign nationals, whereas the other two sites have a much more mixed client-base, it is likely that one factor deterring migrant women from seeking care is xenophobia. By not attending ANC, women with high-risk pregnancies slip through the net and cannot be referred for closer monitoring during childbirth or for an elective Caesarean section. Gumede’s study also found that attendance was especially low among adolescents – another high-risk group – likely due to stigma related to early sexual activity, and concomitant disrespect and abuse by health workers, an issue that has long been a problem for adolescents attending South African public health facilities [16]. Clearly, there are intersecting axes of marginalisation and vulnerability that shape access to care, and further research is needed to document the long-term impact of the anti-migrant sentiment that periodically racks South African cities.

These multiple levels of vulnerability may then be perpetuated into the next generation. Children and adolescents in this setting are affected by a range of social ills: alcohol abuse, violence and negative gender norms are particularly pervasive [17, 18] – highlighting how the social determinants of health play an important role, especially in the HIV epidemic. This framework is useful for focusing attention on how factors such as poor housing quality, high population density, limited access to clean water and sanitation, and the presence of drugs and gangs in the social environment play a critical role in shaping health outcomes [19]. And while these factors are not uniquely urban, they do play themselves out in very particular ways in cities [20]. Others have pointed to a series of “interlinked deprivations” [21] experienced by the urban poor, especially where inequality is as entrenched in the fabric of social life, as it is in South Africa. The everyday reality of experiencing such deprivations are perhaps most evident in the two articles here that consider the impact on young people of the violence that is now virtually endemic in South African cities, using data from the Well-being of Adolescents in Vulnerable Environments (WAVE) study [22, 23]. In an analysis of polyvictimisation and mental health among adolescents in five disadvantaged urban settings across the globe, Mphatso Kamndaya et al. show that even compared to some of the most deprived inner-city areas in the world, the levels of violence in Hillbrow are extreme. Overall, boys and girls in this setting had experienced higher exposure to interpersonal violence than their counterparts in the other five cities and were at greater risk for all mental health indices assessed: depression, suicidal thoughts and posttraumatic stress. Yet the provision of mental health services for this vulnerable population was far from adequate: only 3 of the 500 adolescents interviewed had ever accessed such services or were receiving treatment. Stigma, especially among boys, remained a major obstacle preventing young people from seeking out treatment.

These themes are echoed in the companion piece by Fiona Scorgie et al., which uses qualitative data from the WAVE study to understand the spatial and gendered dimensions of inner-city violence, analysing Hillbrow in terms of a “geography of personal danger and safety”. They ask how adolescent girls and boys in Hillbrow experience violence differently, partly through examining the maps drawn by these young people to illustrate places of relative safety in the neighbourhood. While for girls, the threat of sexual harassment and assault in public spaces emerged as a pervasive threat, boys indicated that they feared local gangs, physical violence, and being drawn into substance-abuse. Home – whether in a shelter or cramped apartment in Hillbrow, boarding with relatives – was sadly not a safe haven. In ways that virtually mirrored girls’ experience of Hillbrow’s public spaces, home was also a gendered space of sexual violence, abuse and neglect. In this inner-city setting, the spectre of the alcohol-HIV nexus was especially evident, and it was concerning that young people living here were so frequently exposed to alcohol use. Without early preventative action, they are poised, in time, to adopt harmful drinking patterns themselves. And yet, there were signs that these young people had developed remarkable resilience – for example, in the ability to ‘reframe’ the challenges of living in Hillbrow in more positive terms – even as they found health and social services woefully inadequate to meet their needs.

The importance of ‘place’ – the physical environment and the range of social meanings it attracts – emerges again as a strong theme in an article that examines substance abuse and HIV prevention in inner-city migrant hostels. Originally created by mining companies to accommodate male workers at minimal cost, these urban, single-sex male hostels were part of an Apartheid strategy to limit the urbanisation of black people from rural *bantustans*. Now largely abandoned by mining companies, the hostels are under rudimentary control of the municipality and mostly occupied by unemployed migrants. Most are overcrowded and in disrepair, while on their periphery, equally crowded informal shack settlements have formed, housing mainly women. Formative research undertaken by the RHRU showed the extent to which hostel and informal settlement residents felt “alienated” from people in the broader city of Johannesburg. This alienation might explain, in part, the harmful alcohol use described in this setting by Braimoh Bello and others, and how it impacts on sexual risk behaviour. Gender played a strong role in shaping these associations: heavy episodic drinking was the strongest predictor of unsafe sexual behaviours in men, particularly of perpetration of sexual violence. Considerably fewer women than men were drinkers, but these women had especially harmful drinking patterns and an even stronger association with unsafe sexual behaviour. The juxtaposition of single-sex male hostels and informal settlements populated largely by women has meant that social connections are often established within drinking venues, and sexual exchanges in these settings are routinely mediated through alcohol. Looking ahead, more rigorous interventions are needed to alleviate alcohol harms, both at community level and on a broader public health scale, for example, through efforts to control the alcohol industry.

Despite the negative consequences associated with migration to the inner-city, there is some cause for hope. Aside from the signs of resilience among adolescents found by the WAVE study, we may extract promising results from community-based, people-centred approaches to intervention design. The question of what it means to incorporate community engagement and take seriously the results of formative research in the design of an urban HIV intervention is tackled in an article by Scorgie and colleagues. Again, the setting is the cluster of inner-city single-sex hostels and surrounding informal settlements described by Bello et al., where residents are mainly economic migrants from other provinces in South Africa; the challenge is how to respond to high rates of HIV transmission in these interlinked communities. Together, the hostels and shack settlements on their margins form part of the geographical and conceptual category of ‘informal urban areas’, which in 2012 registered an HIV prevalence among 15–49 year olds that was double that of ‘formal urban areas’ [24]. But when inviting residents to set out their priorities, it was the issues associated with daily survival that were most pressing. HIV prevention activities therefore needed to be embedded within a broader health and development focus, in which HIV was addressed as part of a nexus of vulnerability, with clear gendered and spatial dimensions.

These disadvantaged urban settings call for supportive policy and planning environments that allow for interventions to be tailored to the local context, and ensure accessibility of healthcare services for marginalised populations. But these may not be enough. As Jo Vearey and colleagues show, community-led initiatives that tap into collaborative partnerships between stakeholders have proven essential in the quest to improve healthcare for cross-border migrants in the inner-city. Progressive legislation may well be in place to guarantee these migrants the right to health services, but in practice, their attempts to access public healthcare and other positive determinants of health in Johannesburg, including housing and employment, are frequently unsuccessful. In recognition of this reality, and as an example of a ‘ground up’ local response to the complex health needs of cross-border migrants, the Johannesburg Migrant Health Forum was formed in 2008. This unfunded, informal working group seeks to hold the state accountable for its constitutional obligation to provide healthcare to non-nationals, bringing together local community members, academics, advocates, healthcare providers and other stakeholders to this end.

Despite the significant impact of the HIV epidemic on inner-city populations, many interventions in this setting have demonstrated success and challenged researchers to ‘think outside the box’. The Hillbrow Health Precinct continues to host one of the largest ARV treatment sites in South Africa, and is a space where researchers and service providers are working together to identify new interventions and improve access for vulnerable populations. Francois Venter et al. report the baseline findings of a randomised controlled trial examining the efficacy and safety of two antiretroviral regimens for treating HIV, and reflect on the criteria and tests used to determine treatment readiness. The authors found that relatively few patients initiating ART had abnormal results on the tests used for screening for ART eligibility, suggesting that the population of HIV-infected people in this setting is relatively healthy. It also brings into question the public health value of renal, hepatic and haematological screening tests prior to ART initiation. In this setting, these tests seem to unnecessarily delay ART initiation while increasing programme complexity and costs, highlighting the need for better and cheaper diagnostics such as point-of-care tests.

When it comes to engaging men in healthcare, it is important to look beyond HIV, of course, and find novel ways to involve them in broader health issues. This challenge was taken up by Admire Chikandiwa and colleagues, who looked at prevalence of anogenital HPV infection and associated disease using baseline data from a cohort of 304 HIV-infected men in Hillbrow. While prevalence of genital and anal Human Papillomavirus (HPV) infection was found to be in the upper range of global estimates, willingness to access services and even interventions that normally have low uptake, such as intra-anal swabbing, were unexpectedly high. The study suggests that high-quality services for men, provided by a male health worker, might overcome the gendered social behaviours, occupational obligations and disinterest in their own health which are believed to account for low male engagement with services. For policy-makers, health planners and providers, these findings underscore the importance of seeking out windows of opportunity to enable services to attract – and retain – these traditionally hard-to-reach populations. They also provide a compelling argument for the extension of HPV vaccination campaigns in this region to include men.

On that note, it is perhaps appropriate to end with a glance to the future and the work being done to end HIV and other epidemics with a vaccine. Lucy Chimoyi and colleagues remind us that most HIV prevention interventions investigated thus far have been targeted at women, who have a higher risk for infection and a compelling need for female-controlled methods of HIV prevention [25–27]. What this means is that men tend to be involved in trials only indirectly, in their role as sexual partners of women, rather than as actual participants. But an effective HIV vaccine is still the best long-term hope for controlling the pandemic and this needs to be tested in both sexes [28]. Chimoyi’s article seeks to fill evidence gaps around the feasibility of recruiting and retaining heterosexual men in an HIV vaccine trial. In a novel trial design, a Hepatitis B Virus vaccine was given as a surrogate for an HIV vaccine (the control group received the vaccine after study completion). The researchers found that against all expectations – given Hillbrow’s reputation as a space where residents are highly transient, but also because of men’s historically low rates of accessing healthcare in the region as a whole – retention of men in the trial was higher than in most similar studies. And men’s motives for enrolling were also somewhat unexpected, with many citing an altruistic desire to help find an efficacious HIV vaccine, along with attributes of the clinical environment: cleanliness, the welcoming attitudes of clinic staff, and being treated by a male health worker. Seemingly obvious and even superficial adjustments to healthcare services, like making services for men more male-centred and staffed by men, can really make a difference to attracting men to arenas where they are traditionally under-represented: HIV trials, but also routine SRH services.

In closing, despite the diversity of focus among articles in this Supplement, some common lessons emerge about managing health in the inner-city. We should not underestimate the impact of migration on the health system, and how migrants new to the city – even those within their own country – experience exclusion. In this region, rapid urbanisation brings both positive opportunity, but also threats to public systems that were not designed to deal with high volumes of people. Both at facility level and in the community as a whole, much more needs to be done to counter the high levels of xenophobia that incapacitate migrants and hinder their access to care. Living in inner-city slums – especially as a member of a marginalised group, such as sex workers and migrants – has a cumulative effect of reinforcing poor health and heightening risk for disease. Structural interventions are needed to address social factors like violence, stigma, legal discrimination and lack of adequate housing, using creative urban planning and interventions that are innovative, rights-based and tailored to specific settings.

We can no longer ignore how social context and ‘place’ shape health and access to services. As a social disease, HIV has provided a powerful lens to focus our attention on the social and structural determinants of health, and the importance of reducing inequalities. The lessons learned from nearly three decades of responding to HIV among marginalised inner-city communities need to be adapted now to tackle other health challenges: this is what it means, surely, for cities to become truly resourceful and resilient. Just as migrants to Johannesburg over the past century have come to the city with hope for a better future, so too can we trust that this setting will offer opportunities to find new solutions: urban citizens in general are resilient and creative. Even in the most deprived areas of the city, networks of social and human resources exist that can be tapped into to produce assets for urban health interventions [20]. Furthermore, taking seriously the input of inner-city residents is essential if health interventions are to have any chance of succeeding. Future programming would benefit from research that builds a better understanding of the risk mitigation strategies developed by vulnerable populations, particularly populations that are under-studied. There is still much to learn from critical analysis of the health needs and healthcare-seeking patterns among men in the inner-city. Ageing, also in the context of HIV, is important yet often overlooked, and it would be good to assess the role of elders in these aspects of health.

It is time for renewed, vigorous and innovative responses to the challenges documented here, many of which are not particularly *new*, but persist nonetheless in undermining the rights of inner-city residents to decent healthcare and decent life chances. Central to this approach must be a commitment to render the city as a whole more inclusive for poor and marginalised communities.
